# An Accessory Protease Inhibitor to Increase the Yield and Quality of a Tumour-Targeting mAb in *Nicotiana benthamiana* Leaves

**DOI:** 10.1371/journal.pone.0167086

**Published:** 2016-11-28

**Authors:** Philippe V. Jutras, Carla Marusic, Chiara Lonoce, Carole Deflers, Marie-Claire Goulet, Eugenio Benvenuto, Dominique Michaud, Marcello Donini

**Affiliations:** 1 Département de phytologie, Université Laval, Québec Quebec, Canada; 2 Laboratory of Biotechnology ENEA Research Center, Casaccia, Rome, Italy; US Naval Research Laboratory, UNITED STATES

## Abstract

The overall quality of recombinant IgG antibodies in plants is dramatically compromised by host endogenous proteases. Different approaches have been developed to reduce the impact of endogenous proteolysis on IgGs, notably involving site-directed mutagenesis to eliminate protease-susceptible sites or the *in situ* mitigation of host protease activities to minimize antibody processing in the cell secretory pathway. We here characterized the degradation profile of H10, a human tumour-targeting monoclonal IgG, in leaves of *Nicotiana benthamiana* also expressing the human serine protease inhibitor α_1_-antichymotrypsin or the cysteine protease inhibitor tomato cystatin *Sl*CYS8. Leaf extracts revealed consistent fragmentation patterns for the recombinant antibody regardless of leaf age and a strong protective effect of *Sl*CYS8 in specific regions of the heavy chain domains. As shown using an antigen-binding ELISA and LC-MS/MS analysis of antibody fragments, *Sl*CYS8 had positive effects on both the amount of fully-assembled antibody purified from leaf tissue and the stability of biologically active antibody fragments containing the heavy chain Fc domain. Our data confirm the potential of Cys protease inhibitors as convenient antibody-stabilizing expression partners to increase the quality of therapeutic antibodies in plant protein biofactories.

## Introduction

Plants present several advantages over microbial expression systems for the production of recombinant proteins, such as the ability to fold complex heteromeric proteins or to perform mammalian-like post-translational maturation of nascent protein backbones [1]. Several proteins of medical interest have been successfully produced in plant systems over the last two decades [2–4], notably including monoclonal antibodies for the diagnosis or treatment of human diseases [5–7]. On the other hand, and although plants have been widely investigated for the production of clinical-grade monoclonal antibodies against human tumours [8], the West Nile virus [9] or the Ebola virus [10], it is only recently that a first plant-made antibody was approved by regulatory bodies for a first-in-human Phase I clinical trial [7].

Transient protein expression in plants such as the widely used host *Nicotiana benthamiana* is a convenient way to quickly produce large amounts of bioactive antibodies. A major drawback of this approach, however, is the presence of non-assembled antibody fragments as a result of proteolytic processing *in planta* [11]. Endogenous proteases are involved in many biological processes, and hundreds of genes coding for these enzymes have been identified in plant genomes [12,13]. Protease activities in plant protein biofactories may lead to partial or complete hydrolysis of recombinant antibody chains in leaf cells or in the leaf apoplast [14, 15], typically leading to the concomitant isolation of full-size antibodies and stable fragments from crude protein extracts following purification [16]. Despite numerous reports on antibody degradation (e.g. [5, 17, 18]), it remains challenging to draw general rules for antibody processing in plants, except for the antibody hinge and nearby regions well known for their high susceptibility to proteolysis [19, 20].

In practice, the host proteolytic machinery may dramatically affect the yield of several recombinant proteins in plant systems [21] and the identification of endogenous protease activities altering the integrity of recombinant IgGs remains a major issue [22, 23]. Protein engineering approaches have been devised to overcome unintended antibody proteolysis *in planta*, involving the removal of protease-susceptible sites by site-directed mutagenesis [24] or the design of stable chimeric antibody variants by the substitution of variable heavy chain domain sequences [18]. Host cell engineering approaches have also been proposed, notably to create protease activity-depleted environments for maturing recombinant proteins [13, 23, 25]. One approach along this line consists of silencing host protease forms using DNA antisense or RNAi sequences [26–28]. Another approach consists of co-expressing accessory protease inhibitors with the protein of interest to inhibit endogenous protease activities *in situ* [29, 30]. Co-secretion of tomato cystatins *Sl*CYS8 or *Sl*CYS9, two Cys protease inhibitors, was shown for instance to improve the accumulation of a transiently expressed diagnostic monoclonal IgG in *N*. *benthamiana* leaves [13, 31]. Similarly, a soybean Ser protease inhibitor secreted by the hairy roots of transgenic tobacco lines was shown to stabilize the light and heavy chains of IgG variants co-secreted in the hydroponic culture medium [32].

Building upon these developments, our objectives in this study were to further document the negative effects of endogenous proteolysis on recombinant antibodies in *N*. *benthamiana* leaves, and to characterize the antibody-stabilizing effects of co-expressed protease inhibitors at the domain sequence level of a promising therapeutic antibody. Tomato cystatin *Sl*CYS8 [33] and human serpin α_1_-antichymotrypsin (α_1_-ACT) [34] were used as accessory inhibitor models for the *in situ* inactivation of Cys and Ser proteases, respectively. H10, a human monoclonal IgG reported to target the tumour-associated antigen tenascin-C [8], was used as a model antibody. The general degradation profile of H10 in *N*. *benthamiana* leaves and a number of protease-susceptible sites in the heavy chain sequence of this antibody have been described recently [11, 20].

## Materials and Methods

### Gene expression vectors

**Fig 1:**
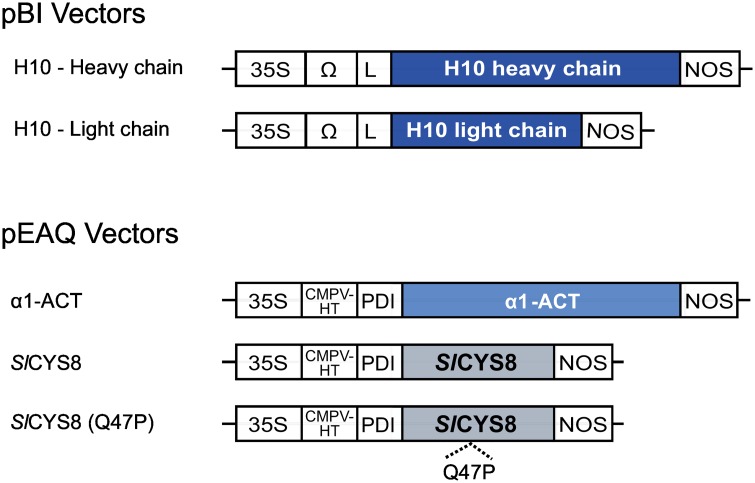
Gene constructs for antibody and protease inhibitor expression in *N*. *benthamiana* leaves. The diagram identifies coding sequences of individual H10 antibody heavy and light chains for insertion in a pBI expression vector, and those of the accessory protease inhibitors α_1_-ACT, *Sl*CYS8 and *Sl*CYS8 inactive mutant Q47P for insertion in a pEAQ vector. All coding sequences were placed under the control of a Cauliflower mosaic virus 35S promoter (35S) and an Ω or *CPMV-HT* translational enhancer sequence. L, signal peptide for antibody chain cellular secretion, from the heavy chain of an embryonic mouse immunoglobulin; PDI, signal peptide of an alfalfa protein disulphide isomerase; NOS, nopaline synthase terminator sequence.

Gene constructs for H10 were previously described and used for transient expression in *N*. *benthamiana* leaves [8]. In brief, DNA sequences encoding the H10 heavy and light chains were assembled with appropriate DNA regulatory sequences into the binary vector pBI-Ω. The constructs included a Cauliflower mosaic virus 35S promoter sequence for constitutive expression, an Ω translational enhancer sequence and the nopaline synthase terminator sequence. The antibody chains were flanked with an N-terminal protein secretion signal peptide derived from an embryonic mouse immunoglobulin HC-encoding gene, finally resulting in two distinct plasmids, pBI-ΩH10HC and pBI-ΩH10LC (Fig 1). Gene constructs for the protease inhibitors were assembled by Golden Gate cloning and assembled into a modified pEAQ vector [35] as described previously [36]. The coding sequences of tomato *Sl*CYS8 (GenBank accession no. AF198390) and human α_1_-ACT (GenBank accession no. J05176) were flanked with the N-terminal signal peptide of a protein disulphide isomerase (PDI) from alfalfa [37], to give the expression vectors pEAQ-*Sl*CYS8 and pEAQ-α_1_-ACT, respectively (Fig 1). A pEAQ vector for Q47P, an inactive form of *Sl*CYS8 produced by site-directed substitution of residue Gln-47 for a proline in the *Sl*CYS8 donor clone, was used as a functional negative control for *Sl*CYS8 *in planta* [38]. All gene constructs were verified by DNA sequencing before plant transfection assays.

### Transient expression in *N*. *benthamiana* leaves

The pBI and pEAQ vectors were maintained in *Agrobacterium tumefaciens* strain AGL1 [39], and the bacterial cultures for transfection assays grown to stable phase in Luria-Bertani medium supplemented with appropriate antibiotics. The bacteria were recovered by gentle centrifugation at 4,000 *g*, resuspended in leaf infiltration medium (10 mM MES (2-[N-morpholino]ethanesulfonic acid), pH 5.6, 10 mM MgCl_2_) and let to grow for 2 to 4 h at 20°C up to an OD_600_ of 0.5. The bacterial suspensions were either vacuum-infiltrated [40] or pressure infiltrated with a needleless syringe [41] in leaves of 42 day-old *N*. *benthamiana* plants. Infiltrated plants were incubated at 20°C in a growth chamber, and their leaves harvested six days post-infiltration for recombinant protein extraction and detection. Bacterial cultures for the H10 antibody were mixed with an equal volume of bacterial culture for either *Sl*CYS8 or α_1_-ACT, or with an equal volume of bacterial culture transformed with the ‘empty’ pEAQ vector. The resulting bacterial mixtures were co-infiltrated with a pEAQexpress bacterial culture at the same density to provide expression of the silencing suppressor protein P19 [35]. All experiments were conducted with three plants of comparable morphological age to allow for statistical analyses.

### Protein extraction and immunoblotting

Infiltrated leaves were harvested as leaf discs representing 160 mg of control-infiltrated tissue, and homogenized by disruption with ceramic beads in a Mini-Bead-beater apparatus (BioSpec, Bartlesville OK, USA). Soluble proteins were extracted in three volumes of phosphate-buffered saline (PBS) buffer, pH 7.3, containing 5 mM EDTA, 0.05% v/v Triton X-100, and the complete protease inhibitor cocktail (Roche Diagnostics, Laval QC, Canada). Leaf lysates were clarified by centrifugation for 20 min at 20,000 *g*, and the extracted proteins resolved by 10% or 12% w/v SDS-PAGE. H10 chains were immunodetected with appropriate antibodies following electrotransfer onto nitrocellulose membranes in 20 mM Tris–HCI transfer buffer containing 152 mM glycine and 20% v/v methanol, after electrophoretic protein separation in reducing or non-reducing conditions. Non-specific binding sites on nitrocellulose membranes were blocked with 5% w/v skim milk powder in PBS buffer containing 0.025% v/v Tween-20, which also served as antibody dilution buffer. H10 heavy and light chains were detected with goat anti-human γ chain horseradish peroxidase (HRP)-conjugated polyclonal antibodies (Sigma-Aldrich, Oakville ON, Canada; Product No. A8419, dilution 1:10,000) and goat anti-human λ chain HRP-conjugated polyclonal antibodies (Sigma-Aldrich A5175, 1:10,000), respectively. After a 1-h incubation at ambient temperature, chemiluminescent signals were revealed using the ECL Advance Western blotting detection kit (GE Healthcare, Baie d’Urfé QC, Canada). The ECL signals were captured on an X-ray film and then scanned for quantification. Densitometric analysis was performed with antibody patterns from three independent biological replicates using the Phoretix 2D Expression software, v. 2005 (NonLinear USA, Durham NC, USA), after numerizing the X-ray films with an Amersham Image Scanner (GE Healthcare).

### Enzyme-linked immunosorbent assays

Immulon 2HB enzyme-linked immunosorbent assay (ELISA) plates (Immuno-Chemistry Technologies, Bloomington MN, USA) for the quantification of H10 heavy and light chains were coated for 1 h at 37°C with freshly prepared leaf protein extracts. Serial extract dilutions were prepared in PBS buffer and a relative standard curve was generated from diluted H10 samples. All dilutions (controls and samples) were performed in a control extract from leaf tissue infiltrated with a mock inoculum so that any unspecific matrix effect was eliminated. The plates were washed three times in PBS buffer, blocked for 1 h at 37°C with 1% w/v casein in PBS buffer (Pierce, Rockford IL, USA), and washed three times again in PBS buffer. The plates were then incubated with either anti-human λ chain HRP-conjugated (Sigma-Aldrich A5175, 1:40,000) or anti-human γ chain HRP-conjugated (Sigma-Aldrich A8419, 1:40,000) polyclonal antibodies in blocking solution for 1 h at 37°C, to detect the H10 light and heavy chains, respectively. Washes with PBS were repeated, and the plates were incubated with the 3,3’, 5,5’-tetramethyl-benzidine (TMB) Sure Blue peroxidase substrate (KPL, Gaithersburg MD, USA). The reaction was stopped by the addition of 1 N HCl, before reading the absorbance at 450 nm. Each sample was assayed in triplicate and antibody concentrations were interpolated from the linear portion of the standard curve. All measurements were made with leaf protein extracts from three independent (biological) replicates.

An activity ELISA procedure to quantify purified H10 was performed by coating m-TNC BCD (provided by Philogen S.p.A., Italy) at a concentration of 20 μg/ml in 1X PBS onto Nunc-Immuno Maxisorp plates, followed by an overnight incubation at 4°C. The plates were blocked with 1% w/v casein in PBS and serial dilutions of purified products were applied to the plates, starting with a concentration of 4 μg/ml (100 μL per well). After washing, anti-human γ chain HRP-conjugated antibodies (Sigma-Aldrich A8419) diluted at 1:5,000 in 1X PBS were added to detect bound H10. After 30 min, enzymatic activity was measured at 405 nm on a microplate reader (TECAN-Sunrise, Groedig, Austria) using 2,2-azino-di-3-ethylbenz-thiazoline sulphonate as substrate (KPL).

### Protein A affinity chromatography

H10 full-size antibody and heavy chain-containing fragments were purified from agroinfiltrated *N*. *benthamiana* plant extracts by protein A affinity chromatography, essentially as described before [16]. Infiltrated leaves collected six days post-infiltration were pooled and homogenized mechanically in 80 ml of protein extraction buffer (1X PBS, pH 7.3) using an Ultra-Turrax homogenizer T25 (IKA, Staufen, Germany). The slurry was filtered through a Miracloth tissue (Sigma-Aldrich), centrifuged twice at 12,000 *g* for 20 min at 4°C, and filtered through 0.45 μm syringe filters (Millipore, Bedford MA, USA) to eliminate fine particles. The clarified supernatant was loaded onto a protein A affinity column (1 ml HI Trap^™^ rProtein A FF; GE Healthcare) previously equilibrated with protein extraction buffer (1X PBS) at a flow rate of 1 ml/min. The column was washed with 10 ml of PBS (10 column volumes), the antibody eluted with 0.1 M citric acid (pH 3.0) and the resulting samples buffered with 1/5 volume of 1 M Tris-HCl, pH 8.0. Protein samples were resolved by SDS-PAGE and stained with Coomassie blue. Antibody-containing fractions were dialyzed using Slide-A-Lyzer Dialysis cassettes (Thermo Fisher Scientific, Monza, Italy) against 1X PBS. Antibody concentrations were determined spectrophotometrically by reading of the absorbance at 280 nm using an Ultrospec 3000 spectrophotometer (Biochrom, Cambridge, UK) [42].

### Mass spectrometry

Purified H10 heavy chain products co-expressed with *Sl*CYS8 were resolved by 12% w/v SDS-PAGE under reducing and non-reducing conditions prior to liquid chromatography tandem mass spectrometry (LC-MS/MS) analysis. Six protein bands detected following Coomassie blue staining, i.e. four from the reducing gels (bands F1 to F4) and two from the non-reducing gels (bands F5 and F6), were carefully excised with a razor blade and further processed for LC-MS/MS analysis. In-gel digestion and MS/MS analysis were carried out at the Proteomics Platform of the Québec Genomics Center (Québec City QC, Canada). Briefly, gel slices were destained, reduced in 10 mM dithiothreitol, alkylated in 55 mM iodoacetamide and treated overnight at 37°C with 125 nM Trypsin Gold (Promega, Madison WI, USA) using a MassPrep Workstation robot (Waters-Micromass, Milford MA, USA). Peptides in the gel matrix were extracted in 2% v/v acetonitrile (Acn):1% v/v formic acid, followed by 50% v/v Acn:1.0% v/v formic acid. The resulting samples were pooled, vacuum centrifuged and resuspended in 7 μl of 0.1% v/v formic acid, of which 2 μl was taken for LC-MS/MS analysis. Peptide samples were separated by online reversed-phase nanoscale capillary liquid chromatography and analyzed by electrospray mass spectrometry. The analyses were performed with an Eksigent Ekspert NanoLC425 apparatus coupled to a 5600+ mass spectrometer (AB Sciex, Framingham, MA, USA), equipped with a nanoelectrospray ion source. Peptide separation took place on nano cHiPLC columns (3u, 120A C18, 15 cm x 0.075 mm internal diameter). The peptides were eluted over 35 min at 300 nl/min, along a 5–35% solvent B (acetonitrile, 0.1% v/v formic acid) linear gradient. Peptide mass spectra were acquired under a data-dependent acquisition mode using the Analyst software, version 1.7. The 20 most intense ions in the 400–1,250 *m/z* range were selected for collisional induced fragmentation with the dynamic exclusion function enabled, an exclusion duration of 30 s, and the relative collisional fragmentation energy set at 35.

### Protein identification

MS/MS peak lists (.mgf sample files) were generated using the Paragon algorithm of ProteinPilot, version 5.0 (AB Sciex, Framingham, MA, USA). The MGF files were analyzed using Mascot (Matrix Science, London, UK; version 2.5.1) set up to search the CP_HomoSapiens_9606_20140317 database (69151 entries) and assuming a protein digestion treatment with trypsin. Mascot data were filtered to obtain fragments with an ion mass tolerance of 0.1 Da and a parent ion tolerance of 0.1 Da. Carbamidomethylation of Cys residues was specified as a fixed modification; Glu->pyro-Glu of the N-terminus, gln->pyro-Glu of the N-terminus, deamidation of Asn and Gln residues, and oxidation of Met residues were specified as variable modifications. The Scaffold program, version 4.4.1 (Proteome Software Inc., Portland OR, USA) was used to validate MS/MS-based peptide and protein identifications. Peptide identifications were accepted if they could be established at greater than 95% probability by the Scaffold algorithm. Protein identifications were accepted if they could be established at greater than 95% probability and contained at least two identified peptides. Protein probabilities were assigned by the Protein Prophet algorithm [43]. Proteins that contained similar peptides and could not be differentiated based on MS/MS spectra were grouped to satisfy the principle of parsimony.

## Results

### H10 antibody accumulation in *N*. *benthamiana* is leaf age-dependent

**Fig 2:**
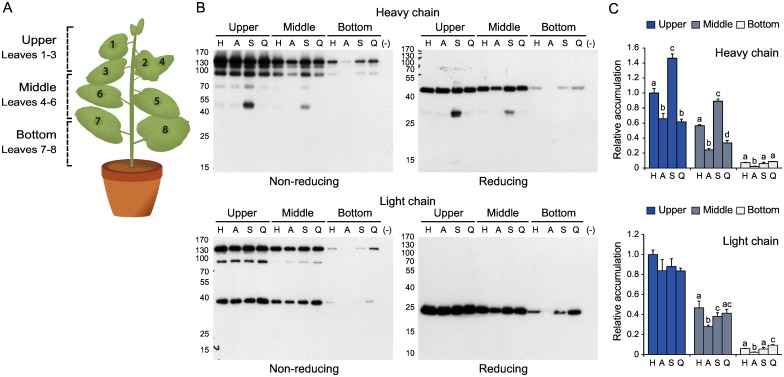
H10 antibody profiles in upper, middle and bottom leaves of *N*. *benthamiana* expressing the antibody alone or in combination with an accessory protease inhibitor. A: Schematic representation of a typical 42 day-old *N*. *benthamiana* plant, with eight leaves arranged in an alternate pattern along the main stem. The leaves were grouped in three categories based on their position on the stem, from the apex down: the 'Upper' leaves corresponding to young growing leaves (Leaves 1, 2 and 3), the 'Middle' leaves corresponding to fully expanded mature leaves (Leaves 4, 5 and 6) and the 'Bottom' leaves corresponding to older–including senescent–leaves (Leaves 7 and 8). B: Representative immunoblots for full size H10 expression. Leaf protein extracts were submitted to SDS-PAGE in reducing or non-reducing conditions, and then electro-transferred onto nitrocellulose membranes for immunodetection. The membranes were probed with an anti-human γ chain HRP-conjugated antibody to detect the heavy chain or with an anti-human λ chain HRP-conjugated antibody to detect the light chain. The position of molecular mass markers is shown on the left (kDa). (–) stands for empty vector-transfected plants (negative controls). C: Quantitative ELISA for H10 heavy and light chains. The two chains were expressed alone (H) or together with α_1_-ACT (A), SlCYS8 (S) or Q47P (Q). Data are expressed compared to the H alone treatment in upper leaves (arbitrary value of 1.0). Each bar is the mean of three independent (biological) replicates ± se. For each leaf morphological zone, bars with different letters are significantly different (post-anova Tukey’s test; *P*<0.05).

The degradation profile of H10 in tobacco and *N*. *benthamiana* leaves was assessed recently using a range of approaches to elucidate the structural determinants of IgG degradation in plant cells and to identify specific protease-susceptible regions in the expressed antibody sequences [20]. We here investigated the impact of leaf age on H10 levels in agroinfiltrated plants by comparing antibody yields in young and older leaves (Fig 2). *N*. *benthamiana* plants with eight leaves arranged in an alternate pattern along the main stem were previously proposed to include three morphological zones encompassing, respectively, young growing leaves (leaves 1, 2 and 3), mature leaves (leaves 4, 5 and 6) and older–including senescent–leaves (leaves 7 and 8) [31]. We numbered the leaves using the same pattern, from the apex down, with the 'Upper' zone corresponding to young leaves, the 'Middle' zone to mature leaves and the 'Bottom' zone to older leaves (Fig 2A). An immunoblot analysis of leaf samples following protein expression showed the overall profile of H10 fragments to be consistent with previous work by Hehle et al. [20] and similar to the fragment profile of another monoclonal IgG, C5-1, expressed in *N*. *benthamiana* [13] (Fig 2B). A significant yield decrease was observed for both the heavy and light chains along the leaf age gradient, despite comparable fragment profiles in all leaf samples suggesting a conserved degradation pattern from the apex to the bottom (*see* samples H corresponding to H10 expressed alone in Fig 2B). The relative amounts of H10 heavy and light chains were assayed by ELISA to further confirm the differential accumulation rates of these polypeptides in upper, middle and bottom leaves (Fig 2C). In accordance with previous work on C5-1 stressing the importance of young leaf tissues on total antibody yields per plant [31], leaves in the upper zone contained high levels of both two chains on protein specific basis, compared to intermediate and very low levels in middle and bottom leaves, respectively (anova; *P*<0.05). These data, along with the limited importance of young leaves on total leaf biomass per plant [31], indicated a negative impact of leaf ageing on overall IgG yield and no correlation between IgG yield and leaf biomass at the whole plant scale.

### Accessory protease inhibitors differentially influence the H10 light and heavy chains

Co-infiltrations were performed with α_1_-ACT, *Sl*CYS8 and the inactive *Sl*CYS8 variant Q47P to assess the impact of accessory protease inhibitors on H10 yield along the leaf age gradient and to link eventual IgG-stabilizing effects with endogenous protease inhibition (Fig 2B and 2C). As previously observed for a number of heterologous proteins co-expressed with other proteins [30], α_1_-ACT had a negative impact on yields of both the heavy and light chains (A lanes on Fig 2B). By comparison, *Sl*CYS8 had little impact on light chain accumulation but a strong positive impact on heavy chain-containing assemblies, especially in upper and middle leaves (S lanes on Fig 2B). A strong band of ~50 kDa was observed in *Sl*CYS8-leaf protein extracts resolved under non-reducing conditions (Fig 2B, upper left panel), similar in size to the intact, non-assembled H10 heavy chain. The higher accumulation of this polypeptide was not reflected, however, in gels run under reducing conditions, where an intense band of 27 kDa and a faint band of 55 kDa were detected instead (upper right panel). No such bands and no positive impact on H10 chain yields were observed in protein extracts from plants expressing the Q47P variant (lanes Q on Fig 2B), confirming the IgG-stabilizing effect of *Sl*CYS8 to be associated with the inhibition of endogenous Cys proteases. ELISA tests were performed to validate the immunoblots and to compare the relative amounts of heavy and light chain products in control and inhibitor-expressing leaves (Fig 2C). As expected, the accumulation of both two chains decreased with leaf age (anova; *P*<0.05). Likewise, *Sl*CYS8 co-expression increased heavy chain content to give overall accumulation levels per plant about 1.5-fold the levels in control plants (post-anova Tukey’s test; *P*<0.05). By comparison, α_1_-ACT and Q47P had a negative impact on heavy chain accumulation (*P*<0.05), to give relative levels of heavy chain products in upper and middle leaves about 50% the levels observed in control plants (Fig 2C). Overall, these data confirmed the potential of Cys protease inhibition to stabilize H10 heavy chains in *N*. *benthamiana* leaves. They also suggested a negative impact of protease inhibitor co-expression on IgG accumulation as observed with other proteins [30,36], strongly compensated by the heavy chain-stabilizing effect of co-secreted *Sl*CYS8 in upper and middle leaves.

**Fig 3:**
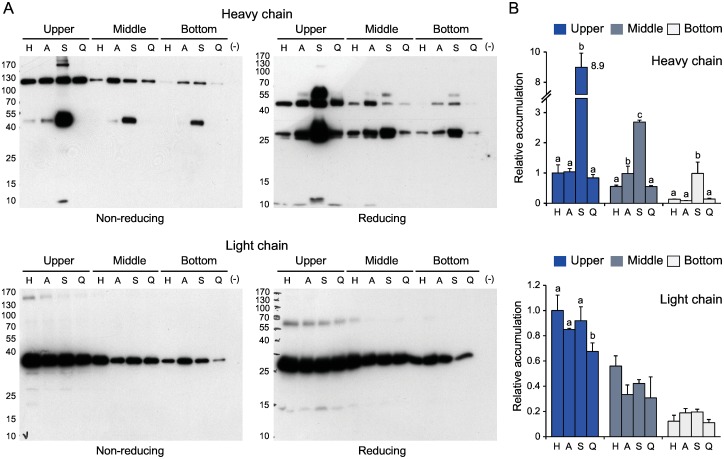
H10 heavy and light chain profiles in upper, middle and bottom leaves of *N*. *benthamiana* expressing either polypeptides alone or in combination with an accessory protease inhibitor. A: Representative immunoblots for the H10 heavy and light chains. Leaf protein extracts were submitted to SDS-PAGE in reducing or non-reducing conditions, and then electro-transferred onto nitrocellulose membranes for immunodetection with an anti-human γ chain HRP-conjugated antibody (heavy chain) or an anti-human λ chain HRP-conjugated antibody (light chain). Numbers on the left show the position of molecular mass markers (kDa). (–) stands for empty vector-transfected plants (negative controls). B: Quantitative ELISA for the H10 heavy and light chains. The two chains were expressed alone (H) or together with α_1_-ACT (A), SlCYS8 (S) or Q47P (Q). Data are expressed compared to the H alone treatment in upper leaves (arbitrary value of 1.0). Each bar is the mean of three biological replicate values ± se. For each leaf morphological zone, bars with different letters are significantly different (post-anova Tukey’s test; *P*<0.05).

The heavy and light chains were expressed separately to further confirm the differential stabilizing effects of *Sl*CYS8 on the two polypeptides (Fig 3). As above, *Sl*CYS8 had a strong positive impact on the accumulation of heavy chain assemblies regardless of leaf age, in contrast with the inactive Q47P mutant showing no effect (Fig 3A). The 50-kDa band detected in *Sl*CYS8-protein extracts under non-reducing conditions (*see* Fig 2B, upper left panel) was also detected in plants expressing only the heavy chain (Fig 3A, upper left panel). Likewise, two bands of ~55 and 27 kDa were detected under reducing conditions together with the 50-kDa band (upper right panel). An ELISA was performed to quantify the chain products co-expressed with *Sl*CYS8 as a function leaf age (Fig 3B). The most significant effect of *Sl*CYS8 was measured for the heavy chain in upper leaves, where a 9-fold yield increase was noted relative to the control compared to 5- and 7-fold increases in middle and bottom leaves, respectively (post-anova Tukey’s test; *P*<0.05) (Fig 3B, upper panel). Overall, this protective effect of *Sl*CYS8 translated into a 7.5-fold yield increase for heavy chain products on a whole plant basis, unlike α_1_-ACT and Q47P both having no net impact on accumulation (*P*>0.05). By comparison and similar to α_1_-ACT and Q47P, *Sl*CYS8 had no impact on light chain accumulation (anova; *P*>0.05) (Fig 3B, lower panel), in accordance with the immunoblot signals (Fig 3A). Taken together, these observations suggest that endogenous proteolytic activities affecting the integrity of H10 assemblies may be mitigated by the co-expression of *Sl*CYS8 in *N*. *benthamiana* leaves, to result in a considerably enhanced accumulation of heavy chain-containing products but having, by contrast, no effect on the light chain.

### *Sl*CYS8 protects specific regions of the H10 heavy chain

**Fig 4:**
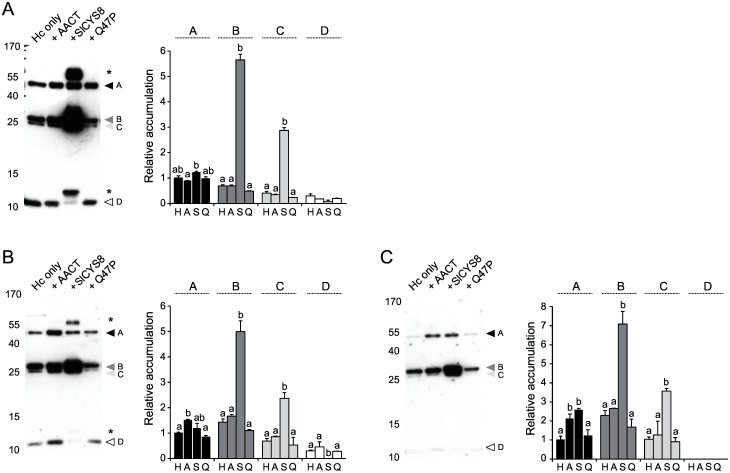
Impact of co-expressed protease inhibitors on the degradation profile of H10 heavy chain domains in *N*. *benthamiana* upper (A), middle (B) and bottom (C) leaves. Protein extracts with the heavy chain expressed alone or co-expressed with protease inhibitors were submitted to SDS-PAGE in reducing conditions and electro-transferred onto nitrocellulose membranes for immunodetection. Heavy chain fragments in plants expressing the heavy chain alone (H) or along with α_1_-ACT (A), SlCYS8 (S) or Q47P (Q) were detected with an anti-human γ chain HRP-conjugated antibody. Arrows A to D point to heavy chain fragments detected in control plant extracts, and the asterisk to additional bands detected in plants co-expressing *Sl*CYS8. Numbers on the left indicate the position of molecular mass markers (kDa). Relative accumulation rates for fragments A to D in the histograms were determined by densitometric analysis of the immunoblot signals. Data are expressed compared to the H alone treatment for fragment A (arbitrary value of 1). Each bar is the mean of three independent (biological) replicates ± se. Bars with different letters are significantly different (post-anova Tukey’s test; *P*<0.05).

Immunoblots were produced following SDS-PAGE in reducing conditions to compare the relative accumulation rates of the most abundant heavy chain fragments in control and protease inhibitor-expressing leaves (Fig 4). Four-fragment patterns were immunodetected in control, α_1_-ACT-expressing and Q47P-expressing plants as protein bands of 50, 27, 26 and 11 kDa, respectively. Similar band patterns were detected in protein extracts of *Sl*CYS8-expressing leaves, except for the addition of a strong band signal at ~55 kDa and the presence of a low-molecular-weight fragment of 13 kDa in place of the 11-kDa fragment (*see* asterisks, left part of Fig 4A and 4B). Relative amounts of the major 50-kDa band (fragment A on Fig 4, corresponding to the full heavy chain), the 27-kDa band (fragment B), the 26-kDa band (fragment C) and the 11-kDa band (fragment D) were assessed in more detail by densitometry to compare the impact of *Sl*CYS8 and α_1_-ACT on each fragment (Fig 4, right panels). Unlike fragment A showing no variation, fragments B and C were found at strongly increased levels in *Sl*CYS8-expressing plants (post-anova Tukey’s test; *P*<0.05), with steady-state levels ~4 to 9-fold the levels observed in control leaves depending on leaf age. By contrast, fragment D disappeared in the presence of *Sl*CYS8 (*P*<0.05) (Fig 4A and 4B), concomitant with the appearance of the slightly larger 13-kDa band suggesting a protective effect for the inhibitor at specific Cys protease-accessible sites on the heavy chain (Fig 4A). By comparison, band patterns in protein extracts of Q47P-expressing plants were similar to those of plants expressing the heavy chain alone or along with α_1_-ACT (*P*>0.05), again confirming a link between the stabilizing effect of *Sl*CYS8 and the inhibition of host Cys proteases.

**Fig 5:**
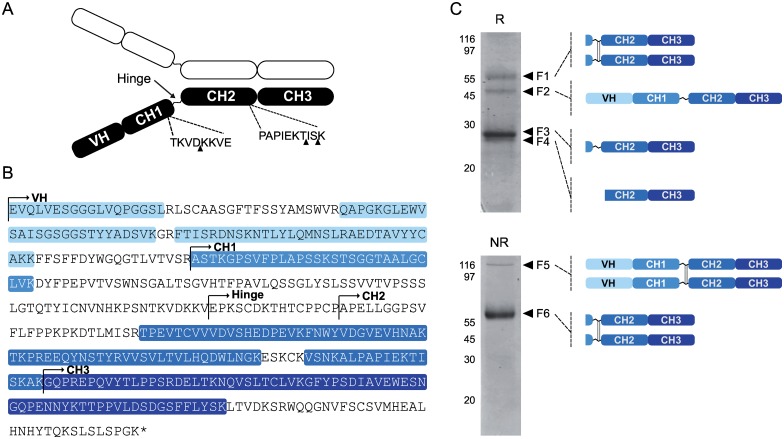
MS/MS characterization of H10 heavy chain fragments isolated from *Sl*CYS8-expressing plants. A: Schematic diagram for the H10 heavy chain, highlighting the variable VH domain, the constant domains CH1, CH2 and CH3, and the hinge region linking CH1 to CH2. Black arrows point to the most important cleavage sites of H10 heavy chain in the *N*. *benthamiana* leaf cell secretory pathway as recently inferred by Hehle et al. [20]. B: Amino acid sequence of H10 heavy chain. Peptide sequences identified by LC-MS/MS (*see* panel C) are shaded in blue. C. Correspondence between MS/MS unique peptide data and heavy chain domain fragments or assemblies detected on Coomassie blue-stained gels following SDS-PAGE in reducing (R) or non-reducing (NR) conditions. Six fragments (bands F1 to F6) detected following electrophoresis and Coomassie blue staining were excised manually and submitted to LC-MS/MS analysis. Schematic representations on the right indicate the composition of each fragment based on MS/MS data. Sequence details for identified unique peptides are given in S1 Table.

Heavy chain fragments in protein extracts from *Sl*CYS8-expressing plants were purified by protein A affinity chromatography and resolved by SDS-PAGE under both reducing and non-reducing conditions to identify regions in the polypeptide protected by the cystatin (Fig 5). N-terminal sequencing work on H10 degradation products in *N*. *benthamiana* leaves recently allowed to identify three protease-susceptible sites in the heavy chain [20], including one site close to the hinge region and two others at the C-terminus of the CH2 domain (Fig 5A). Six major bands consisting of four chain fragments detected following electrophoresis in reducing conditions (bands F1 to F4) and two chain fragments detected following electrophoresis in non-reducing conditions (F5 and F6) were here selected for LC-MS/MS analysis to look at the impact of *Sl*CYS8 on the processing of these putative cleavage sites (Fig 5B and 5C). Detailed information on unique peptides identified for each chain fragment is given in S1 Table, including the relative abundance of heavy chain domains within the detected fragments. A total of 27 different peptides were detected following LC-MS/MS, covering overall ~57% of the whole heavy chain sequence (*see* highlighted sequences on Fig 5B). Of these peptides, eight were specific to the VH domain, eight to the CH2 domain, eight to the CH3 domain and three to the CH1 domain. All heavy chain domains were identified from the unique peptide sequences, to give coverage rates of 66%, 31%, 73% and 64% for the VH, CH1, CH2 and CH3 domains, respectively. Bands F3, F4 and F6 mostly contained Fc domain-associated peptides, similar to band F1 likely corresponding to an Fc domain-containing dimer that remained stable in reducing conditions. Bands F3 and F4 showed a very similar composition, with no peptide specific to the CH1 domain. These two fragments were indeed identical, except for a small difference in molecular weight suggesting differential cleavage in the CH1 domain near the hinge region. Band F5, at about 100 kDa, contained peptides covering the whole chain sequence, in accordance with the natural tendency of this polypeptide to form homodimers. The domain composition of each band as inferred from the MS/MS data is schematized in Fig 5C. Our data suggest overall that *Sl*CYS8 co-expression in *N*. *benthamiana* leaves had little impact on the VH-CH1 region, but a strong protective effect on the constant domains promoting the accumulation of Fc-containing domains.

### *Sl*CYS8 co-expression improves the overall quality of purified H10 preparations

**Fig 6:**
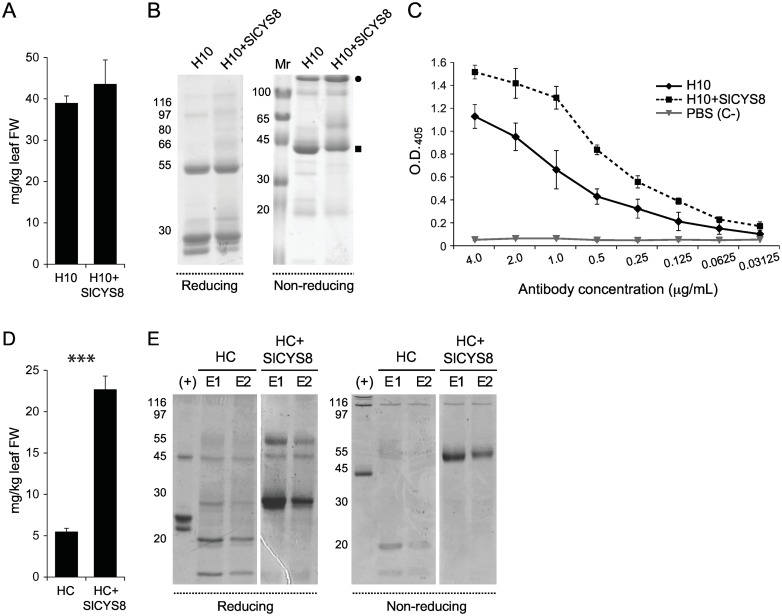
Protein A-based affinity purification of H10 full size antibody and heavy chain fragments from leaf protein extracts of control and *Sl*CYS8-expressing plants. A: Purification yield of H10 protein products expressed alone or along with *Sl*CYS8. Average yields (± se) were calculated from three independent purification rounds. B: Coomassie blue staining of purified full size H10 and H10 fragments following SDS-PAGE in reducing or non-reducing conditions. The closed circle points to full size H10, the closed square to a stable heavy chain-containing fragment. C: Antigen-binding activity of purified H10. The purified antibodies were added to TNC-coated plates at serial dilutions starting from 4 μg/ml. Data are the mean of three independent experiments ± sd. PBS (C–), negative control. (D) Purification yield of H10 heavy chain expressed alone or together with *Sl*CYS8. Average yields (± se) were calculated from three independent purification rounds. Asterisks indicate a highly significant difference between the two purification yields (Student’s t-test; *P*<0.001). (E) Coomassie blue staining of purified H10 fragments following SDS-PAGE in reducing or non-reducing conditions. E1 and E2 show two elution fractions during protein A chromatography. (+), purified H10 used as a reference. On panels B and E, numbers on the left correspond to molecular mass markers (kDa).

H10 full-size antibody and heavy chain-containing fragments were affinity purified from control and *Sl*CYS8-expressing leaves to measure the overall impact of *Sl*CYS8 on antibody product yield and quality (Fig 6). Average protein yield from three independent purification rounds was estimated at 39.0 ± 1.7 mg/kg leaf fresh weight for H10, similar to 43.6 ± 5.8 mg/kg leaf fresh weight for H10 co-expressed with *Sl*CYS8 (Student’s *t*-test; *P* = 0.2578) (Fig 6A). The purified protein products were resolved by SDS-PAGE in reducing conditions to visualize the antibody chains (Fig 6B). IgG preparations from plants co-expressing *Sl*CYS8 included two major bands of ~25-kDa and ~50-kDa following electrophoresis corresponding to the light and heavy chains, respectively. Control plants expressing the antibody alone included the same two bands, along with an extra fragment approximately 2 kDa smaller than the 25-kDa light chain product. Protein separation in non-reducing conditions showed the presence, in both *Sl*CYS8- and control preparations, of a ~150-kDa band corresponding to the intact full-size IgG (closed circle on Fig 6B) and a major fragment at ~44 kDa (closed square). In line with the presence of a smaller (degradation) product in control preparations, densitometric analysis of Coomassie blue-stained gels showed the full-size antibody band in control samples to represent only ~26% of total purified antibody in protein preparations, compared to ~55% in plants also expressing the plant cystatin. These observations indicating a positive impact of *Sl*CYS8 on the quality of the final product were confirmed by an ELISA activity assay against recombinant BCD domains of mouse tenascin-C showing higher antigen binding in H10 preparations from plants co-expressing the inhibitor (Student’s *t*-test; *P*<0.05) (Fig 6C).

Additional protein A purification rounds were conducted with plants transfected to express the H10 heavy chain, alone or in combination with *Sl*CYS8 (Fig 6D and 6E). An average total protein level of 5.5 ± 0.4 mg/kg leaf fresh weight was obtained after purification of the heavy chain expressed alone, compared to a higher purification yield of 22.7 ± 1.6 mg/kg leaf fresh weight for the heavy chain co-expressed with *Sl*CYS8 (Student’s *t*-test; *P*<0.001) (Fig 6D). As seen on Coomassie blue-stained gels following SDS-PAGE in reducing conditions, this 4-fold difference in protein concentration was associated with the occurrence of two abundant protein products of ~55 and ~27 kDa in preparations of *Sl*CYS8-expressing plants, compared to the absence of these protein products and the relative abundance of two degradation fragments at ~20 and ~10 kDa in control plant preparations (Fig 6E, left panel). Major differences in band patterns were also observed in non-reducing conditions, again including the 20- and 10-kDa degradation products in control plants and a strong band signal at ~55 kDa in the *Sl*CYS8-expressing plants (Fig 6E, right panel).

## Discussion

Chinese hamster ovary cells remain the preferred host for the commercial production of monoclonal IgGs but alternative expression systems such as those involving transient expression in agroinfiltrated plants have attracted special attention in recent years [44]. A practical challenge at present to further confirm the potential of plants as bio-factories for therapeutic IgGs is to improve their overall stability by a better control of endogenous proteolysis in host tissues. Plants engineered to express mammalian antibodies generally produce considerable amounts of these proteins on a leaf weight basis, but unintended proteolysis in the cell secretory pathway or later on in the apoplast often reduces the overall yield and quality of the resulting protein products [11]. Human blood-typing antibody C5-1 was reported for instance to undergo restricted proteolysis in *N*. *benthamiana* leaves, visualized as a multiple band pattern following SDS-PAGE in non-reducing conditions and leading to the co-purification of Fab-containing proteolytic fragments together with the full-size antibody following protein A affinity chromatography [13, 31, 45]. Similarly, H10 expressed in tobacco or *N*. *benthamiana* leaves was visualized as several protein bands following non-reducing SDS-PAGE and was purified as a mixture of full-size IgG and stable proteolytic fragments after protein A chromatography [16, 20]. In accordance with these reports, we here detected H10 as a multiple protein band pattern following non-reducing SDS-PAGE in protein extracts of both young and older leaves, encompassing the ~150-kDa full-size IgG and stable fragments of smaller size. As observed with other proteins transiently expressed in *N*. *benthamiana* or tobacco [31, 46], different amounts of antibody product were found in leaf tissue depending on leaf age but the band patterns detected on immunoblots were roughly comparable in all leaf samples. These observations suggest overall a conserved degradation pattern for the H10 antibody in *N*. *benthamiana* despite the previously reported leaf age-dependent increase of endoprotease activities in both the intracellular and apoplastic environments of agroinfiltrated leaves [31]. In practice, these observations also suggest the eventual feasibility of developing mitigation strategies for host protease activities that are useful in all leaves, regardless of their position on the plant.

Antisense DNA, gene silencing and IgG engineering strategies have been described in recent years to elude unintended proteolysis in plant systems, involving the downregulation of host protease expression or the removal of protease-susceptible sites by targeted mutagenesis or domain substitution [18, 24, 27, 28]. Studies have also discussed the potential of protease inhibitors as co-expression partners to enhance the stability of clinically-useful recombinant proteins, including mammalian IgGs [13, 29–32, 36, 47, 48]. In line with these studies and with studies reporting the negative impact of host plant Cys proteases on the integrity of several recombinant proteins in plants [13, 26, 28, 31, 49], we here observed a significant stabilizing effect of tomato *Sl*CYS8 on the H10 antibody. As expected given the reported stability of H10 light chain in *N*. *benthamiana* leaves [16], the stabilizing effect of *Sl*CYS8 was essentially associated with the heavy chain, notably in the CH2–CH3 constant region. From a quantitative standpoint, the cystatin had little impact on total amounts of IgG product purified from leaf extracts following protein A affinity chromatography. It led, on the other hand, to a strong enrichment of the full-size antibody in the purified product to give a relative amount of full-size IgG estimated at 55%, more than twofold the relative amount obtained with control plants. Studies will be welcome in coming years to assess the impact of cystatins on the overall quality of different plant-made IgGs, keeping in mind the similar chain fragment patterns observed for H10 and the anti-HIV mAb 2G12 in *N*. *benthamiana* leaves [20] and the fragment pattern of mAb C5-1 co-expressed with *Sl*CYS8, similar to the pattern here generated for H10 with the same cystatin [31]. Studies will also be welcome to further document the strong stabilizing effect of *Sl*CYS8 on the CH2–CH3 constant region. The practical usefulness of heavy chain Fc domains as fusion protein partners has been discussed in recent years [50, 51], notably to increase the stability of recombinant proteins in plant systems [52] or to facilitate their purification from leaf crude extracts [53].

## Supporting Information

S1 Appendix Raw data for Figs 2, 3, 4 and 6. (XLSX)

S1 Table Complement to Fig 5: H10 unique peptides detected by MS/MS in the heavy chain fragment samples F1 to F6. (PDF)
