# Supplementary material for: An Accessory Protease Inhibitor to Increase the Yield and Quality of a Tumour-Targeting mAb in Nicotiana benthamiana Leaves
Source: PLoS One. 2016 Nov 28;11(11):e0167086. doi: 10.1371/journal.pone.0167086 (PMC5125672; doi:10.1371/journal.pone.0167086)
Supplement: S1 Table — (PDF) [file pone.0167086.s002.pdf]

S1 Table. Complement to Fig 5: H10 antibody unique peptides detected by MS/MS in heavy chain fragment samples F1 to F6.

		No. spectral counts					
**H10 domains**	**Peptides**	**F1**	**F2**	**F3**	**F4**	**F5**	**F6**
**VH**	EVQLVESGGGLVQPGGSLR		2	1		3	1
	QAPGKGLEWVSAISGSGGSTYYADSVK					1	
	FTISRDNSK					1	
	FTISRDNSKNTLYLQMNSLR					1	
	DNSKNTLYLQMNSLR	2	9	1	2	9	2
	NTLYLQMNSLR	2	10	2	2	10	2
	AEDTAVYYCAK		1			1	1
	AEDTAVYYCAKK					1	
	**VH Total**	**4**	**22**	**4**	**4**	**27**	**6**
**CH1**	ASTKGPSVFPLAPSSK	1	6	1		7	
	GPSVFPLAPSSK	1	6			7	1
	STSGGTAALGCLVK		2			3	1
	**CH1 Total**	**2**	**14**	**1**	**0**	**17**	**2**
**CH2**	TPEVTCVVVDVSHEDPEVK					1	3
	FNWYVDGVEVHNAK	4	8	14	10	15	16
	TKPREEQYNSTYR	2	2	2	3	4	4
	EEQYNSTYR	4	4	4	4	5	4
	VVSVLTVLHQDWLNGK		1	5	3	5	7
	VSNKALPAPIEK	3	1	3	3	4	3
	ALPAPIEK	12	5	58	21	60	74
	ALPAPIEKTISK	2	2	2	2	1	1
	**CH2 total**	**27**	**23**	**88**	**46**	**95**	**112**

S1 Table. Continued.

		No. spectral counts					
**H10 domains**	**Peptides**	**F1**	**F2**	**F3**	**F4**	**F5**	**F6**
**CH3**	AKGQPREPQVYTLPPSR			2	1	2	
	GQPREPQVYTLPPSR	3	2	4	2	4	1
	GQPREPQVYTLPPSRDELTK	3	2	10	9	13	6
	EPQVYTLPPSR	18	18	19	17	16	23
	EPQVYTLPPSRDELTK	18	20	27	22	30	26
	NQVSLTCLVK	1	2	2	2	3	3
	GFYPSDIAVEWESNGQPENNYK						4
	TTPPVLDSDGSFFLYSK	2	4	9	6	7	12
	**CH3 Total**	**45**	**48**	**73**	**59**	**75**	**75**
	**Total spectral counts**	**78**	**107**	**166**	**109**	**214**	**195**
	**Proportion VH/total**	**5,1%**	**20,6%**	**2,4%**	**3,7%**	**12,6%**	**3,1%**
	**CH1/total**	**2,6%**	**13,1%**	**0,6%**	**0,0%**	**7,9%**	**1,0%**
	**CH2/total**	**34,6%**	**21,5%**	**53,0%**	**42,2%**	**44,4%**	**57,4%**
	**CH3/total**	**57,7%**	**44,9%**	**44,0%**	**54,1%**	**35,0%**	**38,5%**
